# Bioprinting on Organ-on-Chip: Development and Applications

**DOI:** 10.3390/bios12121135

**Published:** 2022-12-06

**Authors:** Maria Anna Chliara, Stavroula Elezoglou, Ioanna Zergioti

**Affiliations:** 1 School of Applied Mathematics and Physical Sciences, National Technical University of Athens, 15780 Zografou, Greece; 2 Institute of Communication and Computer Systems, 15780 Zografou, Greece; 3 PhosPrint P.C., Lefkippos Technology Park, NCSR Demokritos Patriarchou Grigoriou 5’ & Neapoleos 27, 15341 Athens, Greece

**Keywords:** organ-on-chip, 3D bioprinting, biofabrication, cell patterning, microfluidics

## Abstract

Organs-on-chips (OoCs) are microfluidic devices that contain bioengineered tissues or parts of natural tissues or organs and can mimic the crucial structures and functions of living organisms. They are designed to control and maintain the cell- and tissue-specific microenvironment while also providing detailed feedback about the activities that are taking place. Bioprinting is an emerging technology for constructing artificial tissues or organ constructs by combining state-of-the-art 3D printing methods with biomaterials. The utilization of 3D bioprinting and cells patterning in OoC technologies reinforces the creation of more complex structures that can imitate the functions of a living organism in a more precise way. Here, we summarize the current 3D bioprinting techniques and we focus on the advantages of 3D bioprinting compared to traditional cell seeding in addition to the methods, materials, and applications of 3D bioprinting in the development of OoC microsystems.

## 1. Introduction

Nine out of ten drug candidates fail drug approval during phase 1, 2, and 3 clinical trials, after they have entered clinical studies [1,2]. Issues at any developmental stage can incur huge financial costs and even approved drugs can lead to health problems, resulting from inaccurate preclinical models [3]. The traditional 2D and 3D cell cultures fail to replicate the microenvironment and features of living organs that are critical for their function while they remain principally dependent on time-consuming and costly animal studies [4]. Simultaneously, animal experimentation raises ethical questions, is poorly predictive of human outcomes, and proven unreliable across a wide category of disease areas [5]. Additionally, the U.S. EPA will prevent conducting and funding mammal experiments until 2035 [6], while supporting alternative methods to animal testing [7]. Thus, there is a critical need for more accurate and precise technology to recreate the therapeutically relevant features of an organ in relation to interventional drugs including examining drug delivery and real-time monitoring of the cell and tissue response to a particular stimulation [3].

To fill this gap, the organ-on-chip technologies have been rapidly evolving in the convergence of bioengineering and the science of microfluidics [8]. These microfluidic devices are called «chips» because they were originally fabricated using microfabrication techniques inherited from the manufacturing of computer microchips [9]. The most recent methods that enable the creation of 3D multi-scale mold cavities for the production of microfluidic devices apt for organs-on-chips, include laser stereolithography, laser materials processing on a micrometer scale, electroforming, and micro-injection molding [10].

Despite the growing popularity of the OoC devices, there are still some difficulties that need to be resolved. A major challenge of current OoC systems is the low-throughput character of cell introduction [11]. The initial preparation of OoC systems and the injection of cells is often still a rather manual process. Cell suspensions or cell-loaded hydrogels are pipetted into individual inlet ports and pumped to the desired culture site. Standardization and automation are critical, but complicated due to the importance of sterile cell handling, avoidance of stress, and short time windows. Additional challenges include the correct scaling of organ and tissue sizes as well as cell numbers that must be considered so that active cell ratios are physiologically relevant, and responses to stimulations are accurate [12]. Other difficulties include liquid handling, material compatibilities, monitoring systems, and parallel experimentation [11]. 

The methods of 3D bioprinting of cells, tissues, and organs have been rapidly expanding, leading to partial standardization of the procedure. More specifically, in order to print a complex 3D biological structure with multiple functional, structural, and mechanical components and properties, different combinations of two basic approaches are likely to be needed: biomimicry, a strategy of making identical reproductions of the cellular and extracellular components of a tissue or organ, and autonomous self-assembly, where cellular components of a developing tissue produce their own ECM components, appropriate cell signaling, and autonomous organization and patterning to yield the desired biological microarchitecture and function [13]. The advantages of bioprinting and how it will be applied in order to enhance the efficiency of OoC as well as to overcome some of the crucial challenges that these devices face, are discussed further below.

## 2. 3D Bioprinting Techniques

Bioprinters have been rapidly developing since 1984 [14]. In the last decade, bioprinting setups have been used for printing bioengineered tissues and organs [13,15,16,17]. Basic bioprinting techniques can be divided into two main approaches: nozzle-based methods and light-assisted methods based in optical setups [18]. Other methods include micro-valve bioprinting and acoustic bioprinting, the latter being also an emerging technology for high resolution cell printing [19].

### 2.1. Nozzle-Based Methods for Bioprinting

#### 2.1.1. Inkjet Bioprinting

Inkjet bioprinting is a nozzle-based method where a bioink solution is physically manipulated to create droplets. This type of printer uses gravity, pressure, and the mechanical properties of the bioink solution to eject droplets onto a suitable receiving substrate [20]. 

Based on the different printing strategies, inkjet printing can be categorized into continuous-inkjet (CIJ) and drop-on-demand (DOD). The second inkjet printing strategy, DOD, where droplets are only generated when required, is generally preferred over CIJ for manipulating bioinks. To accomplish this, pressure pulses are generated by thermal [21], piezoelectric [22], acoustic or electrostatic actuators [23]. Droplets can also be generated by using electrohydrodynamic jet bioprinting where high voltage is applied between an extremely small nozzle and the substrate to accumulate the bioink ions towards the substrate [20].

#### 2.1.2. Micro-Extrusion Bioprinting

Micro-extrusion bioprinting is also a nozzle-based method where the bioink solution is forced out of a micro-extrusion head, towards the formation of continuous filaments of biomaterial.

Bioprinting, via micro-extrusion, is most commonly achieved by dispensing a temperature-controlled, usually viscous, bioink with the use of pneumatic [24,25] or mechanical actuators such as piston [26] or screw [13,27]. For the material deposition in 3D space, a relative motion of head and stage in x, y, and z axes is applied [13]. High resolution is succeeded by adjusting the material density and depositing lines of single cells [28].

#### 2.1.3. Freeform Reversible Embedding of Suspended Hydrogels (FRESH)

FRESH is a novel nozzle based technique that is being utilized for constructing 3D structures embedded in suspended hydrogels and is applicable in biomedical device fabrication [29]. The formation of the 3D constructs is achieved by the 3D space movement of the nozzle in a rheologically-adjusted hydrogel bath.

### 2.2. Light-Assisted Methods for Bioprinting 

#### 2.2.1. Laser-Induced Forward Transfer

During the laser-induced forward transfer (LIFT) technique, a pulsed laser beam focuses on a bioink containing donor causing a local and rapid evaporation of the liquid, resulting in a cavitation bubble. When the latter collapses, the surface tension of the bioink brakes and squirts towards the receiving substrate. 

The most common donor setups consist of tape or quartz slides coated with a biocompatible dynamic release layer (DRL) material. The bioink that is transferred from the donor surface can be deposited in a variety of receiving substrates [30,31,32,33,34].

#### 2.2.2. Stereolithography and Digital Light Processing

Stereolithography (SLA) and digital light processing (DLP) use similar mechanisms for 3D bioprinting [35].

In SLA printing, a laser light is applied on the surface of a bioink photosensitive material to form a solidified layer. After the first layer has solidified, the platform rises, and a second layer is photocrosslinked. This is repeated until the complete shape is printed [36]. During DLP, the light is projected onto the photosensitive biomaterial through digital light mirrors instead of a point [37]. 

#### 2.2.3. Micro-Molding Bioprinting

The construction of well-controlled cell-laden hydrogels, in shape and size, using micro-molding techniques, is an outgrowth of traditional soft lithography technologies. In this method, cells are suspended in a hydrogel precursor solution containing a photoinitiator and the mix is poured and crosslinked via UV radiation into the desirable shape [38]. Micro-molding can also be achieved through thermal polymerization [39].

#### 2.2.4. Two-Photon Polymerization

Two-photon polymerization is a growing method, relying on micro-optics, to bioprint three-dimensional micro- and nano-structures. Specifically, it is a photochemical process, during which a femtosecond laser beam, focused by a high-numerical aperture objective on a small volume of photosensitive material, excites the photoinitiators through two-photon absorption which concludes to photopolymerization [40,41]. 

#### 2.2.5. Tomographic Volumetric Bioprinting (TVB)

Tomographic volumetric additive manufacturing (VAM) allows the fabrication of complex geometries with hollow channels in scattering materials, such as cell-laden hydrogels, in tens of seconds [42,43,44,45]. The material polymerization relies on the precise delivery of multiple tomographic light projections. To achieve the correct 3D light dose deposition in the material, the light patterns used for photo-polymerization must propagate inside the resin, accounting for the light distortions by the gel.

#### 2.2.6. Filamented Light (FLight) Bioprinting

Flight biofabrication utilizes the phenomenon of optical modulation instability of filament light beams inside a photosensitive biomaterial, for bioprinting highly aligned hydrogel microfilaments [46].

### 2.3. Micro-Valve Bioprinting

Micro-valve bioprinting offers controlled deposition of materials via a layer-by-layer manufacturing approach through the synchronized ejection of biomaterials and cells from different printheads. A typical microvalve-based bioprinting system comprises a moving in 3D space robotic platform and an array of multiple electromechanical microvalve printheads [47].

### 2.4. Acousting Bioprinting

For studies that require precise deposition of single cells, acoustic-based bioprinting is a viable method to transfer picolitre quantities of the medium or hydrogel that encapsulates an individual cell in a droplet. The liquid medium is ejected by focused acoustic waves, generated by a piezoelectric actuator, on the donor surface [19,20].

## 3. Advantages of Bioprinting in OoC Devices

**Figure 1:**
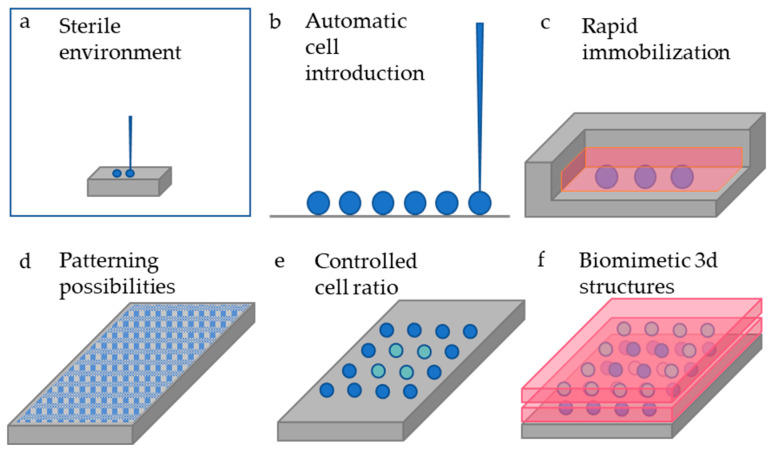
(**a**) Bioprinters and microfluidic devices can be installed in a sterile environment, (**b**) cells are being introduced automatically instead of manually in microfluidic culture chambers, (**c**) some bioprinting techniques offer direct immobilization of cells in desirable positions, (**d**) complex printing patterns can be generated, (**e**) better monitoring of printed cell ratio is achieved, (**f**) biomimetic 3D tissue structures can be printed in vitro.

The bioprinting methods that were briefly described above, can be utilized for resolving some of the major challenges in developing high throughput OoC systems (Figure 1).

- In most cases, the introduction of cells in microfluidic devices is preformed manually via pipette. Knowing that laboratory personnel is one of the greatest sources of contamination [48], by installing bioprinters inside sterile environments, human interference would be minimized which will lead to less exposure to contaminating agents.
- Bioprinting techniques will enhance the automatic introduction of cells in microfluidic devices for developing less time-consuming experiments with higher reproducibility [49] than those where the cells are introduced via pipette.
- Rapid immobilization of cells, achieved through bioprinting, supports direct introduction of liquid flow. Normal cells, when seeded with a pipette, take 6–8 h to develop attachment proteins, hence when seeded in microfluidic devices, they are usually left overnight to reach adherence, resulting in serious time delays before adding culture media flow.
- Pertinent to a specific organ or tissue, types of cells can be patterned only via bioprinting [50,51,52,53], and placed in specific positions inside the microfluidic devices. Bioinks can also be utilized to recapitulate the complex vasculature system and create biological barrier patterns [54,55].
- To replicate physiological cell functions in vitro, it is necessary to simulate cell communications [56] by introducing the desired cell to cell ratios with great precision. Bioprinted techniques can be utilized to deposit a range of cells per droplet, depending on the printing conditions, even with resolutions down to one cell per droplet [19,57], which offers consequential ratio control.
- High levels of biomimicry can be achieved. The greatest benefit of 3D bioprinting is the ability to digitally fabricate the tissue of interest and reproduce the 3D physical structure through automated techniques and at resolutions not possible with traditional photolithography techniques [19,58,59,60].
- Traditional introduction of cells in OoCs or in cell culture plates, with the use of pipettes or pumps, stands as a less expensive cell introduction method; however, it is not suitable for cell patterning, instant immobilization or for achieving high levels of biomimicry.

## 4. Applications of Bioprinting in OoC 

Three-dimensional printing techniques applied in advanced organ-on-a-chip devices can serve as ideal tools for enhancing the biological value of disease models and drug assays. Numerous studies for organ-on-chip models that included bioprinting, are being published with an increasing rate [54,61,62,63]. In this section, we report, discuss, and summarize recent advances in bioprinting applications for organ-on-a-chip models (Table 1. Summarized applications of 3D bioprinting for developing organ-on-chip models).

### 4.1. Blood Vessels and Vascular Microenvironments

**Figure 2:**
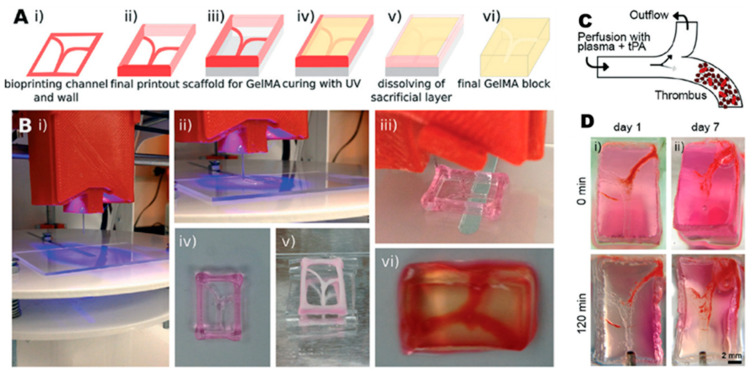
Three-dimensional bioprinted vasculature network to model thrombosis on a chip. (**A**) Three-dimensional bioprinting process patterning schematic. (**B**) Illustration of the experimental procedure step by step according to A. (**C**) Representation of the microfluidic setup. (**D**) Time-lapse photographs of the thrombosis clot at day 1 (i) and day 7 (ii). Reprinted and edited with permission granted by Copyright Clearance Center, Inc. (“CCC”). Published in *Lab on a Chip* journal, by the Royal Society of Chemistry, Vol 16, by Y.S. Zhang et al., “Bioprinted thrombosis-on-a-chip” [65].

**Figure 3:**
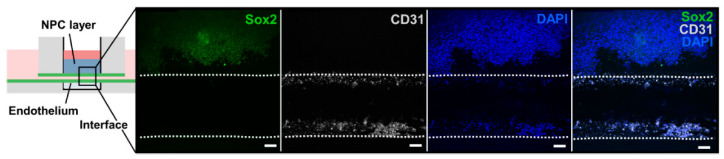
Bioprinted tissue-on-chip with vascular-like channel. Confocal images of hiPSC-NPCs encapsulated within ELP-RGD bioprinted on top of a channel seeded with HUVECs and cultured for 5 days. Scale bars represent 50 mm. Reprinted with permission granted under CC-BY license (https://creativecommons.org/about/cclicenses/ (accessed on 30 October 2022)), published in *Biomaterials*, a section of the journal *Frontiers in Bioengineering and Biotechnology*, Vol 8, by D.F.D. Campos et al., “Bioprinting Cell- and Spheroid-Laden Protein-Engineered Hydrogels as Tissue-on-Chip Platforms” [67].

The modeling of bioengineered cardiac tissues and organ models remains a major challenge due to the structure of the native myocardium [64]. In 2016, Y.S. Zhang et al. [65] fabricated a human thrombosis-on-a-chip with the 3D printed method of micro-molding along with a sacrificial printed layer that was dissolved after bioink’s polymerization. The bioink was a solution of GelMA hydrogel which was later mixed with fibroblasts. The endothelialized micro-channels, after being coated and incubated with HUVECs, were injected with human blood mixture for replicating the formation of clots. This biomimetic human thrombosis-on-a-chip offers us the opportunity to conduct in vitro studies targeting the recapitulation of thrombosis and its variations, such as fibrosis, at a level that exceeds the already existing comparable studies (Figure 2). Later, in 2019, M. Abudupataer et al. used a cell-laden gelatin (GelMA) based hydrogel bioink, printed inside a PMMA CNC drilled microfluidic chip, to construct a vessel-on-a-chip. Endothelial cells, smooth muscle cells and fibroblasts were printed via micro-extrusion method and UV-induced crosslinking [66]. This bioprinted vessel-on-chip model can simulate different types of vessels in vivo with changing cell types and flow parameters. Only one year later, D.F.D. Campos et al. published a study for bioprinting vascularized in vitro tissue-on-chips [67] (Figure 3). Protein-engineered ELP-RGD hydrogels, loaded with cell aggregates, were dispensed by DOD and micro-extrusion bioprinting in chip devices. These endothelialized channels demonstrated distribution of endothelial cells along the entire lumen of the channel.

### 4.2. Brain

**Figure 4:**
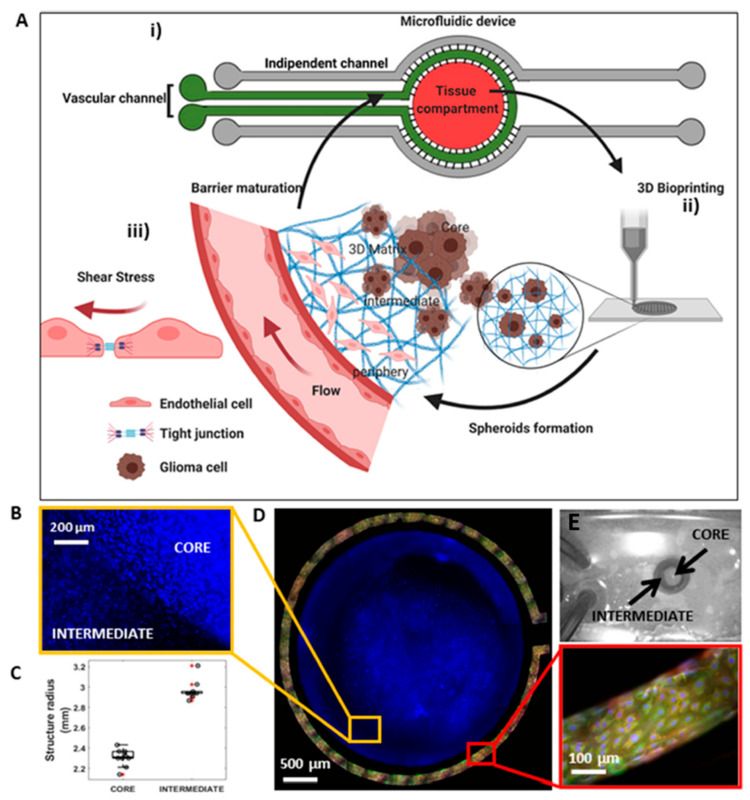
Three-dimensional bioprinted vascularized glioblastoma-on-a-chip. (**A**) Schematic of (i) the microfluidic device, (ii) the bioprinting process and (iii) the flow alongside the embedded tissue. (**B**) Intermediate and core interface (**C**) Scatter data points for structure’s radius. (**D**) Fluorescent images of bioprinted tissue and vascular vessel. (**E**) Photograph of the glioblastoma-on-a-chip. Reprinted with permission granted by Copyright Clearance Center, Inc. (“CCC”). Published in *Advanced Therapeutics* by Wiley-VCH GmbH, Vol 4, by G. Silvani et al., “3D-Bioprinted Vascularized Glioblastoma-on-a-Chip for Studying the Impact of Simulated Microgravity as a Novel Pre-Clinical Approach in Brain Tumor Therapy”, Figure 1 [74].

In the last decade, several organ-on-chip studies, associated to the blood–brain-barrier have been published [68,69,70]. In the cases of disease, the function of the blood–brain barrier that protects the brain is defective, such as in glioblastoma which is the most common brain cancer [71]. In 2019, H.G. Yi et al. bioprinted a human glioblastoma-on-a-chip to identify possible personalized responses to chemotherapy [72]. Patient-derived cells were encapsulated in BdECM and 3D printed via a layer-by-layer hybrid, inkjet, post-crosslinking method [73]. The chip consisted of a combination of permeable and impermeable materials to create an oxygen gradient imitating the central hypoxia of the cancerous tissue. This 3D print-based approach of a glioblastoma-on-a-chip enables a rather fast establishment of a ready-to-test platform for treatment testing [72]. Another study of a glioblastoma-on-a-chip, as a preclinical model, was presented in 2021 by G. Silvani et al. for testing the tumor responses to microgravity [74] (Figure 4). The vascularized tissue construct was a result of two bioinks combined. Brain endothelial cells, encapsulated in GelMA fibrin were bioprinted in a torus shape (intermediate) following the deposition of glioblastoma cell-loaded GelMA–Alginate, in the middle of the ring (core). Overall findings indicate that in the absence of micro-gravitational fields, the invasion and aggregation of glioblastoma cells is inhibited [74]. 

### 4.3. Gut

**Figure 5:**
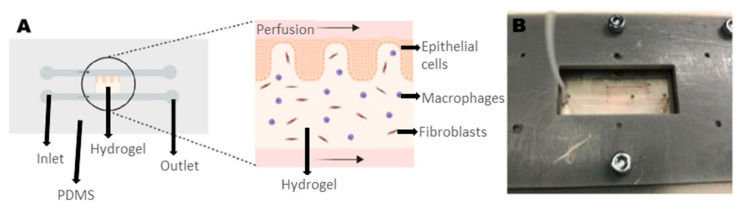
(**A**) Bioprinted gut-on-a-chip model. (**B**) Photograph of the chip and holder, with perfused medium. Reprinted and edited with permission granted under CC-BY license. Published in a Final Degree Project for Biomedical Engineering Degree at the University of Barcelona by E.B. García under the supervision of E.M. Fraiz, “Bioprinted gut-on-a-chip to mimic the small intestinal mucosa” [76].

Gut-on-a-chip in vitro models allow serious improvement in the research field concerning physiology, pathology, and pharmacology of gastrointestinal diseases [75]. The bioprinted gut-on-a-chip presented by E.B. García, as a final degree project in Universitat de Barcelona, offers several advantages compared to existing non-3D bioprinted models [76]. It targets into replicating the epithelial barrier, the lamina propria, and their function in the immune system, adding complexity and increasing the functionality of the model. A custom SLA-based 3D bioprinter was employed for fabricating cell-encapsulating GelMA–PEGDA hydrogels, resembling native tissue, which were later also embedded in an SLA 3D printed microfluidic device (Figure 5). 

### 4.4. Heart

**Figure 6:**
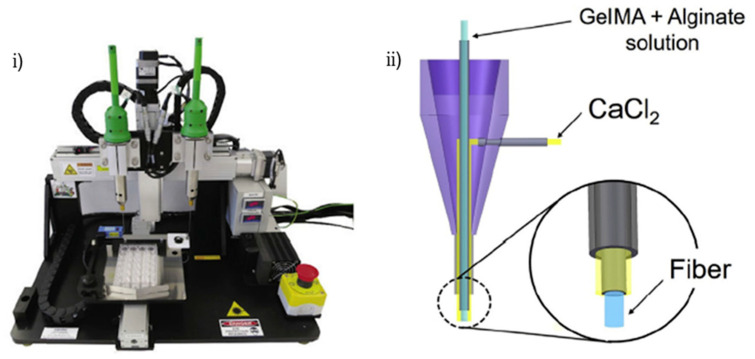

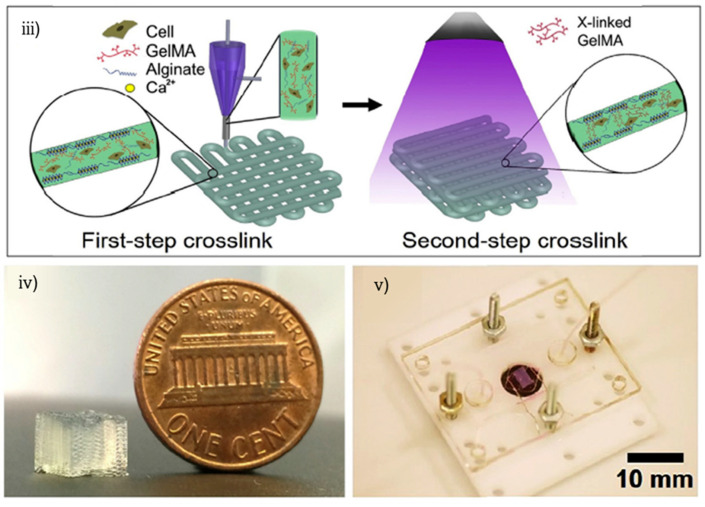
Bioprinted heart-on-a-chip in a microfluidic (**i**) commercial bioprinter Organovo Novogen MMX, (**ii**) printing nozzle schematic, (**iii**) crosslinking process schematic, (**iv**) photograph of a bioprinted cubic microfibrous scaffold (6-mm edge length), (**v**) photograph of the bioreactor with an embedded bioprinted scaffold. Reprinted and edited with permission granted by Rights and Permissions (ELS), all rights reserved. Published in *Biomaterials* by Elsevier. Authors: Y.S. Zhang et al. Title: “Bioprinting 3D microfibrous scaffolds for engineering endothelialized myocardium and heart-on-a-chip” [77].

The modeling of cardiac tissues and organs remains a major challenge due to the structure of the myocardium. In 2016, Y.S. Zhang et al. proposed a novel 3D bioprinting-based procedure for bioengineering endothelialized myocardial tissues [77]. They used a commercial bioprinter, integrated with a custom-made syringe nozzle system connected with a syringe pump, to 3D pattern bioinks. The bioinks mainly consisted of alginate and gelatin-methacryloyl (GelMA) solutions which were later mixed with a suspension of HUVECs. The scaffolds were seeded with cardiomyocytes and then placed in a PDMS microfluidic bioreactor connected with a peristaltic micro pump to support long term viability of the embedded tissue as well as drug screening studies (Figure 6). The combination of bioprinting, microfluidics, and stem cells in the endothelialized myocardium-on-a-chip platform [77] would potentially provide a fundamental technology for the development of next-generation human organ models for the construction of not only healthy and diseased myocardial substitutes, but also the ones that will be utilized in personalized medicine research.

### 4.5. Kidney

In 2016, K.A. Homan et al. reported a study that combines bioprinting, 3D cell culture, and organ-on-a-chip concepts to create a 3D convoluted renal proximal tubule (PT), segment of a nephron, composed of a perfusable open lumen that possesses a programmable architecture, which can support high levels of heterogeneity [39]. The cavities were fabricated via micro-molding, cell-laden ECM, which consisted of fibrinogen, gelatin, and two enzymes, with the support of a sacrificial extrusion-printed layer. Human proximal tubular cells and other types of cells were perfused in the final chip construction to form a tight epithelial monolayer on the tubule walls. These bioengineered proximal tubules-on-chip present significantly improved epithelial morphology and functional properties, compared to the same cells grown on 2D controls with or without perfusion.

### 4.6. Liver

Several applications of bioprinted liver-on-chips have already been reported [78,79], but there are still various issues concerning the recapitulation of liver tissues due to the complex microenvironment of the latter.

**Figure 7:**
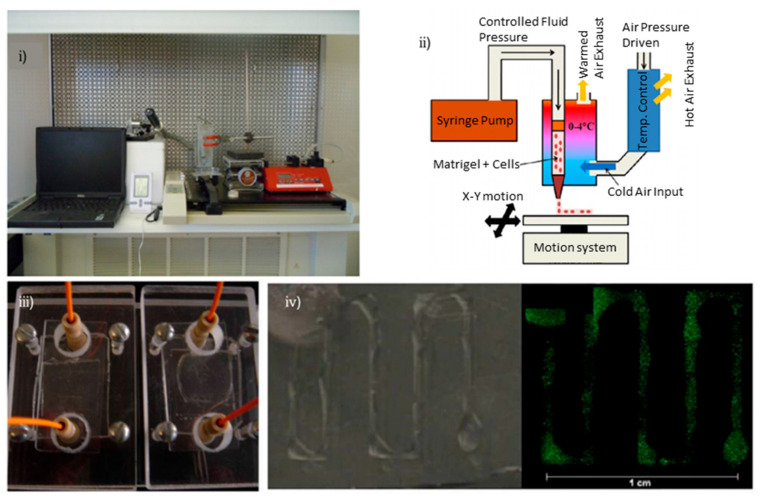
Bioprinted liver-on-chip for drug conversion and radiation protection studies: (**i**) photograph and (**ii**) schematic of the temperature-controlled printing system, (**iii**) microfluidic chip containing cell-laden liver constructs, (**iv**) printed cells. Reprinted and edited with permission granted by Copyright Clearance Center, Inc. (“CCC”). Published in *Biofabrication* by IOP Publishing Ltd. Authors: J.E. Snyder et al. Title: “Bioprinting cell-laden Matrigel for radioprotection study of liver by pro-drug conversion in a dual-tissue microfluidic chip” [81].

In 2008, R. Chang et al. presented a novel at the time of study about the 3D bioprinting of alginate-encapsulated hepatocytes, in a microfluidic platform, by employing a multiple nozzle bioprinting system, in order to develop an in vitro pharmacokinetic model [80]. Three years later, J.E. Snyder et al. used a temperature and motion-controlled extrusion, syringe-based, dispensing system to bioprint human hepatic carcinoma cells and human mammary epithelial cells, encapsulated in a Matrigel solution, on a hybrid PDMS–glass chip [81] (Figure 7). The target of their experiments in the custom dual-tissue chip, was to investigate pro-drug conversion and liver radioprotection.

**Figure 8:**
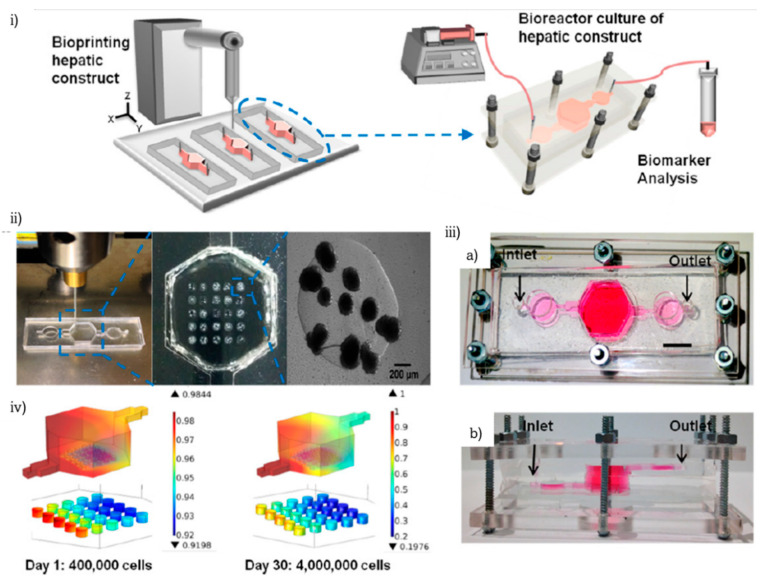
A liver-on-a-chip platform for long-term 3D culture of HepG2/C3A spheroids for drug toxicity assessment: (**i**) schematic of the hepatic bioreactor setup, (**ii**) bioprinting GelMA hydrogel-based hepatic construct within the bioreactor, (**iii**) (a) top and (b) side view of the bioreactor-chip assembly, (**iv**) oxygen concentration gradient. Reprinted with permission granted by Copyright Clearance Center, Inc. (“CCC”). Published in *Biofabrication* by IOP Publishing Ltd. Authors: N.S. Bhise et al. Title: “A liver-on-a-chip platform with bioprinted hepatic spheroids” [82].

Bioprinted liver-on-chip research attracted a lot the interest of the scientific community during 2016, resulting in fruitful outcomes. N.S. Bhise et al., employed a piston-assisted micro-extrusion bioprinter to pattern hepatic spheroid laden GelMA based bioink on a PDMS–PMMA custom chip [82] (Figure 8). The response of the liver-on-a-chip platform to certain treatments was comparable to animal and other in vitro models.

**Figure 9:**
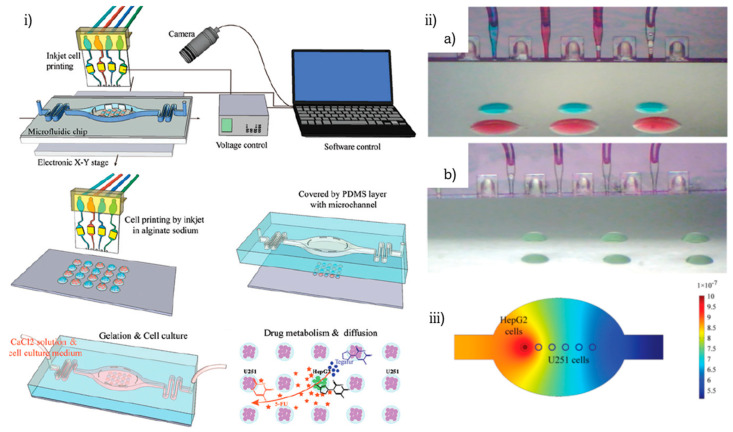
Precise cell patterning for in-chip detection of drug metabolism: (**i**) schematic representation of the experimental setup and process, (**ii**) inkjet printing on (a) hydrophilic and (b) hydrophobic glass surface, (**iii**) modeling of drug metabolism and diffusion from HepG2 cells to U251 cells in the microfluidic device. Reprinted and edited with permission granted by Copyright Clearance Center, Inc. (“CCC”). Published in *Analyst*, by the Royal Society of Chemistry, Vol 141. Authors: J. Zhang et al. Title: “A novel approach for precisely controlled multiple cell patterning in microfluidic chips by inkjet Printing and the detection of drug metabolism and diffusion” [83].

**Figure 10:**
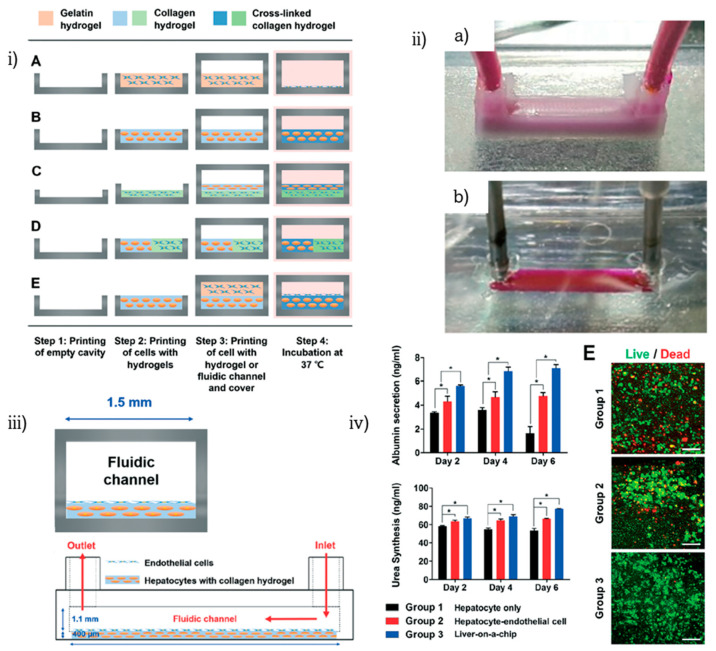
Liver-on-chip in one-step fabrication: (**i**) 3D bioprinting procedure for different organs-on-chips, (**ii**) microfluidic chip from (a) PCL and (b) PDMS, (**iii**) side view and vertical section schematic of the chip, (**iv**) liver function analysis and cell viability (* *p* < 0.05). Reprinted and edited with permission granted by Copyright Clearance Center, Inc. (“CCC”). Published in *Lab on a Chip*, by the Royal Society of Chemistry, Vol 16. Authors: H. Lee and D.W. Cho. Title: “One-step fabrication of an organ-on-a-chip with spatial heterogeneity using a 3D bioprinting technology” [84].

Another innovative method for developing bioprinted liver-on-chip platforms was published the same year by J. Zhang et al. when they applied inkjet bioprinting for depositing hepatoma and glioma cells on glass substrates that were subsequently bonded on fabricated PDMS chips [83] (Figure 9). A cell viability gradient was observed during drug metabolism and diffusion co-culture experiments. Later the same year, H. Lee and D.W. Cho et al. managed to construct an organ-on-chip platform in on-step production, via the pneumatic micro-extrusion printing technique [84] (Figure 10). Five chips were fabricated by utilizing different depositing combinations of cells and ECMs with application to a liver-on-chip system by bioprinting hepatocellular carcinoma and endothelial cells encapsulated in gelatin- and collagen-based hydrogels. The outcome of this research proves that liver function is improved on the 3D bioprinted liver-on-a-chip.

**Figure 11:**
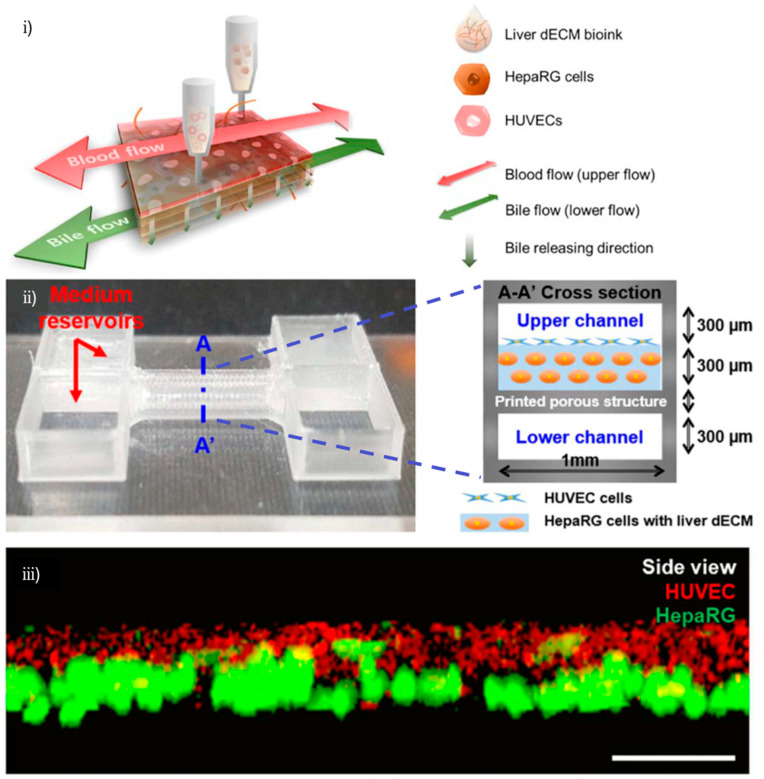
Three-dimensional printing and modeling of liver-on-chip microenvironment for in vitro testing: (**i**) schematic 3D bioprinted layer by layer representation; (**ii**) cell-printed 3D liver-on-a-chip microfluidic device with dual fluidic feature; (**iii**) analysis of cell positioning after cell printing (side view of the channel). Reprinted and edited with permission granted by Copyright Clearance Center, Inc. (“CCC”). Published in *Biofabrication* by IOP Publishing Ltd. Authors: H. Lee et al. Title: “Cell-printed 3D liver-on-a-chip possessing a liver microenvironment and biliary system”, Figure 1, Figure 3 and Figure 4 [85].

In 2019, H. Lee et al. created a liver-on-chip platform with a biliary system, by integrating liver cell types and ECMs, in a custom microfluidic chip, by means of a nozzle-based 3D bioprinting technique [85] (Figure 11). They managed to prove that the function of the chip was superior to 2D or 3D cultures and showed a sensitive drug response. Specifically, a platform was constructed using poly as a structural material on PMMA plates and biomaterials such as gelatin and liver dECMs and HUVECs were printed using a layer-by-layer process.

### 4.7. Lung

**Figure 12:**
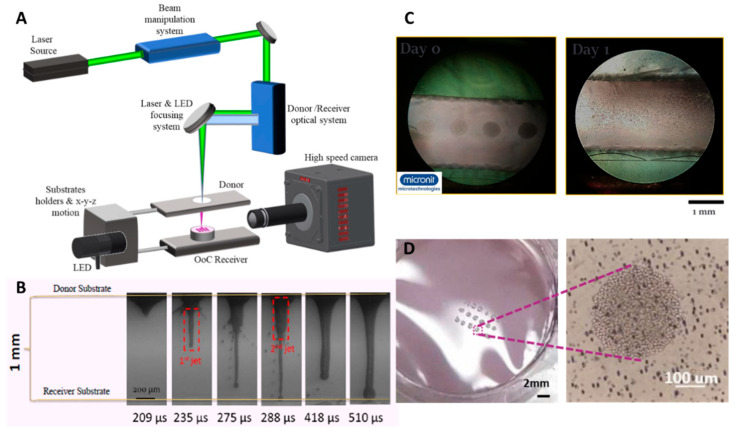
Three-dimensional bioprinting lung cancer cell line inside microfluidic platform for the investigation of possible migrations towards lymph node cells. (**A**) Schematic representation of laser-induced forward transfer (LIFT) bioprinting technique for organ-on-chip applications. (**B**) Jetting analysis during 3D bioprinting using a high-speed camera analysis. (**C**) Bioprinted LLC cells inside a commercially available microfluidic chip https://www.micronit.com/ (accessed on 7 April 2022). (**D**) Bioprinted LLC cells inside OoC’s chamber. Unpublished data from S. Elezoglou et al.

In 2022, S. Elezoglou et al. [34] used laser-induced forward transfer, as a 3D bioprinting technique, to deposit lung cancer cells in an organ-on-chip platform for lung cancer migration studies. The bioink used, was high concentration (75.000 cells/μL) lung cancer cells (Lewis Lung Carcinoma, LLC cell line), which was printed inside a chamber of two different platforms and the cells were observed to be proliferating for a couple of days (Figure 12). At the setup, a high-speed camera was integrated for parallel study of the bioink’s jetting mechanism during 3D bioprinting on the organ-on-chip platform (Figure 12). This study shall constitute a preliminary investigation of artificial tissues inside the OoC before moving to human cell deposition.

### 4.8. Ovaries

A bioprinted ovary-on-a-chip platform study was published in 2020 by Y.S. Choi et al. [86]. Ovarian aggregates were encapsulated in gelatin methacrylate (GelMA) hydrogel and bioprinted in a PCL microfluidic chip that was continuously perfused for 15 days. This bioprinted ovary-on-a-chip platform may provide the first steps for more physiologically relevant and predictive models of ovarian study and disease.

### 4.9. Placenta

**Figure 13:**
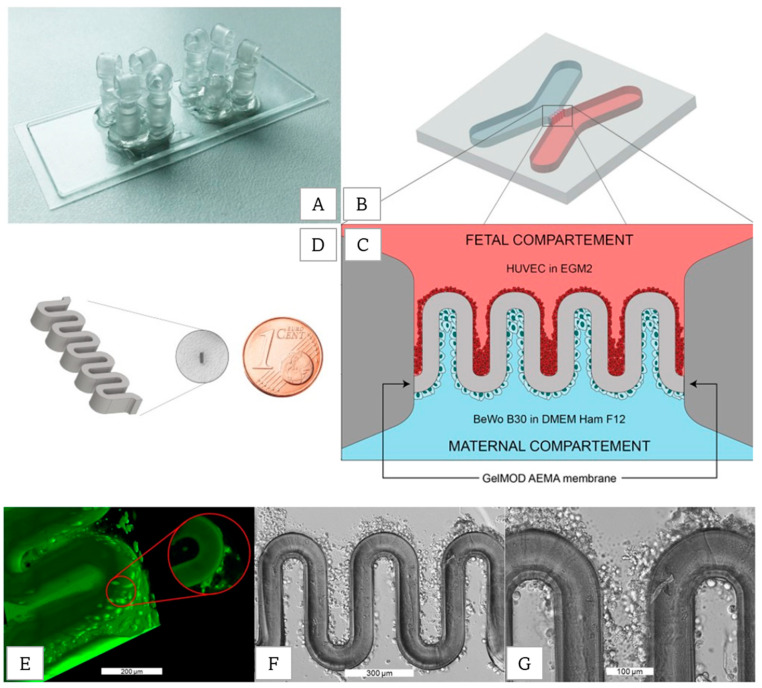
Patterning of ECM biomimetic placenta barrier within a microfluidic device. (**A**) Photograph of the microfluidic chip. (**B**) Schematic illustration of the chip and (**C**) placenta barrier. (**D**) Barrier size relative to a cent. (**E**) Fluorescence images show the cell layer formation of HUVEC on the barrier wall. (**F**,**G**) Cell Layer adhered to the walls. Reprinted and edited with permission granted under CC-BY-NC license. Published by Whioce Publishing Pte. Authors: D. Mandt et al. Title: “Fabrication of biomimetic placental barrier structures within a microfluidic device utilizing two-photon polymerization” [55].

In 2018, D. Mandt et al. applied the two-photon polymerization bioprinting method to pattern placental barrier structures within a microfluidic device [55] (Figure 13). The 3D semi-permeable hydrogel structure, bioprinted in a custom microfluidic chip, enhanced cell adherence and allowed selective transport of substances between the two chambers.

### 4.10. Urothelium

**Figure 14:**
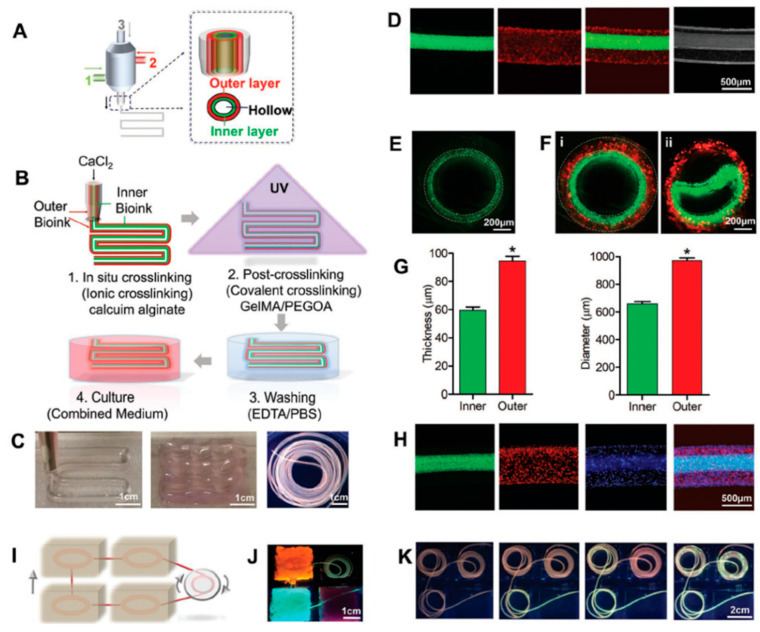
(A) Schematic illustration of the extrusion nozzle and (**B**) bioprinting process of the hollow tubular multilayered tissues. (**C**) Photographs of the bioprinted cannular tissues. (**D**) Fluorescent longitudinal images of the tubes and the cross-section of a (**E**) one-layered and (**F**) double-layered tissue. (**G**) Diameter and thickness of inner and outer layers of the bioprinted tube. (**H**) Fluorescent microscopy images showing bioprinted tri-layered hollow tubes. (**I**) Schematic and (**J**) photograph of the perfusion among tissues. (**K**) Images of the perfusable tissues. Reprinted with permission granted by Copyright Clearance Center, Inc. (“CCC”). Published in *Advanced Materials* by WILEY-VCH Verlag GmbH & Co. KGaA. Authors: Q. Pi et al. Title: “Digitally Tunable Microfluidic Bioprinting of Multilayered Cannular Tissues” [87].

A novel approach for single step bioprinting of solenoid, perfusable urothelial tissues was presented in 2018 by Q. Pi et al. [87] (Figure 14). An extrusion-based bioprinting system was applied to fabricate the multilayered hollowed tubular tissues by simultaneously depositing human urothelial cells and human bladder smooth muscle cells, encapsulated in a custom bioink. The bioprinted constructs exhibited sustained viability and displayed similar to human tubular tissue characteristics, while permitting continuous perfusion of liquids, over a period of two-week experiments.

### 4.11. Tumor in General

Since 2016, it has been known that the bioprinting approach for fabricating biomimetic tumor-on-a-chip platforms shows great promise in accelerating cancer research [88]. These microfluidic-based tumor models that require successful dynamic culture of the artificially engineered tumor tissues, in specifically designed bioreactor chips that replicate tumor microenvironment and physiology, allow precise evaluation of drug toxicity and efficiency [89,90,91]. Current advances in bioprinting technology could enhance cancer treatment and tumor-on-chip throughput character, the first of which still having shortcomings in many areas due to a tumor’s tendency to metastasize, a high recurrence rate, and drug resistance development [90].

**Table 1:** Summarized applications of 3D bioprinting for developing organ-on-chip models.

Reference	Organ/Tissue	Bioprinting Technique	Bioink	Aim	Outcome
Y.S. Zhang et al. [65]	Vascular thrombosis	Micro-molding	Fibroblast mixed with GelMA, HUVECs	Fabrication of an in vitro platform for potential therapeutics to treat thrombosis	Encapsulation of fibroblasts in GelMA demonstrated potential cell migration to clot
M. Abudupataer et al. [66]	Vessel	Micro-extrusion	GelMA, HAECs, HASMC, NIH/3 T3	Stimulate different types of vessels and blood flows	Cellular coculture system based vessel-on-a-chip model with a continuous flow
D. F. D. Campos et al. [67]	Vascular tissue	DoD and Micro-extrusion	ELP, engineered hydrogels, hiPSC-NPCs	Tissue models, di-rectly dispensed onto endothelialized on-chip platform	Compatible bioprinting techniques with single cell suspension and spheroid aggregates of breast cells
H.G. Yi et al. [72]	Glioblastoma	Inkjet	BdECM, patient derived cells	Treatment testing	Establishment of a glioblastoma-on-a-chip platform
G. Silvani et al. [74]	Glioblastoma	Micro-extrusion	GelMA, GBM cells, Endothelial cells	Test of brain tumor responses to microgravity	Absence of micro-gravitational fields, inhibited invasion, and aggregation of glioblastoma cells
E.B. García et al. [76]	Gut	SLA	GelMA, PEGDA, cell-laden	Replicate gut epithelial barrier and lamina propria	No migration observed in gut-on-a-chip
Y.S. Zhang et al. [77]	Myocardium	Micro-extrusion	GelMA, HUVECs	Fabrication of en-dothelialized my-ocardium	Endothelial cells bioprinted within microfibrous hydrogel scaffolds, migration towards peripheries, layer of confluent endotheliumobserved
K.A. Homan et al. [39]	Proximal tubule	Micro-molding	Gelatin fibrinogen	Create 3D human renal proximal tubules in vitro	2-month maintenance in perfusable culture
R. Chang et al. [80]	Hepatocyte tissue	Nozzle-based	Alginate-encapsulated hepatocytes	Development of in vitro pharmacokinetic model	Nonfluorescent prodrug, metabolized by the liver chamber which produced an effluent fluorescent metabolite
J.E. Snyder et al. [81]	Liver	Micro-extrusion	HepG2, M10, Matrigel	Drug conversion and radiation protection of living liver tissue analogs	Observed radiation shielding in the dual-tissue microfluidic system caused by the 2-cell type interaction
N.S. Bhise et al. [82]	Liver	Piston micro-extrusion	GelMA, Hepatic spheroids (HepG2,C3A)	Assembly of a biomimetic liver-on-a-chip platform	In situ monitoring of culture environment, viability after 30 days, responsive to treatment
J. Zhang et al. [83]	Liver	Inkjet	HepG2, U251, alginate hydrogel	Detection of drug metabolism and diffusion	Viability gradient observed during drug metabolism and diffusion
H. Lee and D.W. Cho [84]	Liver	Pneumatic micro-extrusion	HepG2, HUVECs, Gelatin, Collagen	One step liver-on-chip fabrication	Established new micro-engineering method for organ-on-chip to overcome drawbacks
H. Lee et al. [85]	Liver	Nozzle-based	dECM, HUVECs	Fabrication of biomimetic liver-on-chip with biliary system	Function of chip superior to 2D or 3D cultures, sensitive drug response
S. Elezoglou et al. [34]	Lung	LIFT	LLC cell line	Using LIFT Bioprinting technique to deposit high concentration cells in organ-on-chip platform	Preliminary studies of bioprinting lung cancer cells, optimization studies about LIFT bioprinting inside OoC.
Y.S. Choi et al. [86]	Ovary	Not mentioned	Mouse ovarian aggregates, GelMA	Ovary-on-a-chip platform development for ovarian endocrine function in vitro	Physiologically relevant hormonal production
D. Mandt et al. [55]	Placenta	Two-photon polymerization	GelMOD- AEM,	Pattern placental barrier	Versatile biomimetic on chip barrier structure establishment
Q. Pi [87]	Urothelial tissue	Micro-extrusion	hSMCs, HUVECs, HBdSMCs, GelMA	Single step cannular tissues circumferentially multilayered	Sustained viability, similar to human characteristics, 2-week continuous perfusion
Q. Hamid et al. [92]	Tumor	Piston microxtrusion	HepG2, MDA-MB-231	Maskless fabrication techniques for cell-laden microfluidics development	Fabrication system eliminates the limitations of conventional photolithography
X. Cao et al. [93]	Tumor	Nozzle-based	GelMA, PEGDA/PEGOA	Bioprinting of blood and lymphatic vessels	Permeability parameters of bioprinted blood and lymphatic vessels could be controlled by precisely tuning the bioink’s composition
M. Xie et al. [94]	Tumor	Inkjet	GelMA, MDA-MB-231	3D tumor array chip (TAC) fabrication for drug testing	3D-TAC has potential to become a widely applied standard 3D drug screening system

**Figure 15:**
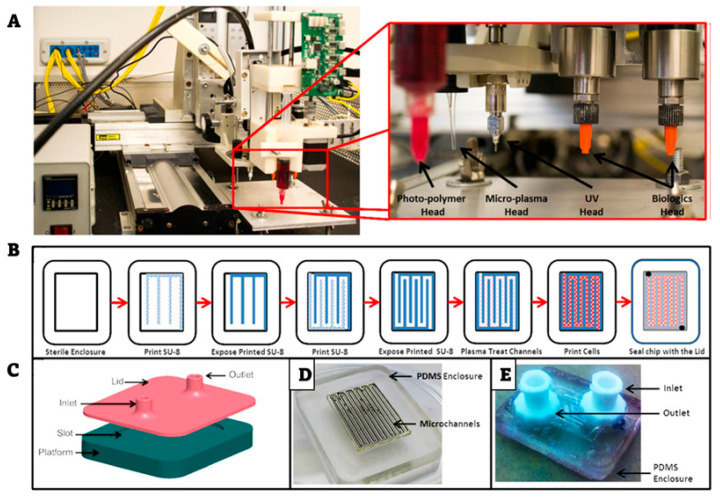
(**A**) Annotated photograph of the fabrication system. (**B**) Schematic representation of the process. (**C**) Illustration of the PDMS enclosure. (**D**) Reprinted and edited with permission granted by Copyright Clearance Center, Inc. (“CCC”). Photograph of the fabricated channels on the PDMS enclosure. (**E**) Photograph of the completed cell-laden microfluid. Published in *Biofabrication* by IOP Publishing Ltd. Authors: Q. Hamid et al. Title: “Maskless fabrication of cell-laden microfluidic chips with localized surface functionalization for the co-culture of cancer cells” [92].

In 2015, Q. Hamid et al. published a novel study where they used a piston-based extrusion bioprinting nozzle, integrated in a fabrication system which also included photopolymer, micro-plasma, and UV heads [92] (Figure 15), in order to bioprint different types of cancer cells in a PDMS microfluidic device (Figure 15). Three years later, X. Cao et al. presented their work on a tumor-on-a-chip platform with a bioprinted pair of blood and lymphatic vessels [93]. A nozzle-based bioprinting system was employed for the printing of the vessel constructs, which were communicating through a cancer cell-laden hydrogel. Later, in 2020, M. Xie et al. published a bioprinted tumor array chip, for drug screening applications [94]. Electrodynamic jet bioprinting was utilized for the deposition of array patterned, hydrogel-encapsulated, cancer cells on a transparent conductive membrane, following comprehensive viability and drug screening experiments on the bioprinted tumor array chip.

## 5. Summary/Conclusions

Three-dimensional bioprinting is an emerging technology that is progressively being applied in the biomedical field. In OoC, state-of-the-art technology in the latter field, 3D bioprinting assists in the throughput activity with tissue engineering (TE) and biofabrication. In this work, the main advantages of 3D bioprinting, as a cell introduction technique in OoC, are highlighted, in comparison with traditional manual cell seeding. Specifically, for fabricating large-scale artificial tissues and organs, extrusion-like or light-projected polymerization bioprinting methods are more appropriate, due to their ability to print higher volumes and higher viscosity biomaterials. On the contrary, for applications where additive manufacturing and higher precision patterning is required, droplet/jet generation-like bioprinting is more suitable. The importance of introducing 3D bioprinting in OoCs is based on the rapid fabrication of organs and tissues, with primary biomaterials and patient-derived and stored cells. Biofabricated samples placed and cultured in microfluidic platforms lead to dependent-free, human biopsy and animal study, OoCs.

## 6. Future Directions

During the last decades, the interplay of multiple research fields, such as biology and engineering, unwrapped the innovative technology of organ-on-chip. The recent integration of 3D bioprinting techniques in organ-on-chip technology has a huge impact on overcoming existing difficulties in traditional in vitro investigations. One of the biggest potential outcomes of organ-on-chip technology is personalized medicine and customized drug discovery. Neither animals, nor humans have the same immune system, hence a possible treatment or vaccine might not be entirely effective or even proven harmful. By constructing artificial tissues and organs inside microfluidic platforms, which are able to function and mimic in vitro, the natural microenvironment of each individual 3D bioprinting is the key to automate the research procedure of personalized medicine. The combination of these two technologies will lead to the production of customized devices, which may offer accurate diagnosis, direct therapeutic services, and individualized drug testing that will target not only treatment, but also disease prevention. The future of the integration of 3D bioprinting techniques in organ-on-chip platforms is to build a whole functional artificial organism or a multi-organ combination, in vitro. Body-on-chip or human-on-chip is the next step in biofabrication, which is directly connected to personalized medicine. Bioprinting methods which offer high throughput character, high reproducibility, and avoid the human factor by automation of the process, will make the practice οf this intricate technology easier. For the commercialization and the fabrication of completely sterilized and user-independent microfluidic platforms, the one-step fabrication of a printed organ-on-chip has already been discussed, including all biomaterials and bioinks needed for its construction. The ideal future outcome of the combination of 3D bioprinting techniques and organ-on-chip technology is the utilization of bioprinted, biopsy-derived, specifically selected cells, placed in a microfluidic platform to target personalized therapy investigation.

## Data Availability

Not applicable.
